# Variation of Bacterial Community Diversity in Rhizosphere Soil of Sole-Cropped versus Intercropped Wheat Field after Harvest

**DOI:** 10.1371/journal.pone.0150618

**Published:** 2016-03-02

**Authors:** Zhenping Yang, Wenping Yang, Shengcai Li, Jiaomin Hao, Zhifeng Su, Min Sun, Zhiqiang Gao, Chunlai Zhang

**Affiliations:** 1 College of Agriculture, Shanxi Agricultural University, Taigu, P. R. China; 2 College of Life Science, North China University of Science and Technology, Tangshan, P. R. China; 3 College of Food Science and Engineering, Shanxi Agricultural University, Taigu, P. R. China; 4 Shanxi Academy of Agriculture Science Sorghum Research Institute, Jinzhong, P. R. China; East Carolina University, UNITED STATES

## Abstract

As the major crops in north China, spring crops are usually planted from April through May every spring and harvested in fall. Wheat is also a very common crop traditionally planted in fall or spring and harvested in summer year by year. This continuous cropping system exhibited the disadvantages of reducing the fertility of soil through decreasing microbial diversity. Thus, management of microbial diversity in the rhizosphere plays a vital role in sustainable crop production. In this study, ten common spring crops in north China were chosen sole-cropped and four were chosen intercropped with peanut in wheat fields after harvest. Denaturing gradient gel electrophoresis (DGGE) and DNA sequencing of one 16S rDNA fragment were used to analyze the bacterial diversity and species identification. DGGE profiles showed the bacterial community diversity in rhizosphere soil samples varied among various crops under different cropping systems, more diverse under intercropping system than under sole-cropping. Some intercropping-specific bands in DGGE profiles suggested that several bacterial species were stimulated by intercropping systems specifically. Furthermore, the identification of these dominant and functional bacteria by DNA sequencing indicated that intercropping systems are more beneficial to improve soil fertility. Compared to intercropping systems, we also observed changes in microbial community of rhizosphere soil under sole-crops. The rhizosphere bacterial community structure in spring crops showed a strong crop species-specific pattern. More importantly, ***Empedobacter brevis*,** a typical plant pathogen, was only found in the carrot rhizosphere, suggesting carrot should be sown prudently. In conclusion, our study demonstrated that crop species and cropping systems had significant effects on bacterial community diversity in the rhizosphere soils. We strongly suggest sorghum, glutinous millet and buckwheat could be taken into account as intercropping crops with peanut; while hulled oat, mung bean or foxtail millet could be considered for sowing in wheat fields after harvest in North China.

## Introduction

Rhizosphere soil is a complex and dynamic element in the field ecological system. Although microbes including bacteria and fungi are very small parts of soil composition, they play important parts in nitrogen, phosphorus and sulfur cycling as well as ecosystem functions. They contribute to soil structure stabilization, organic residue accumulation, nitrogen fixation and toxin removal [1–4]. Microbial species and their populations in the rhizosphere soil also contribute considerably in maintaining health of the crops [5,6]. They are regarded as one of the most sensitive biological indicators for monitoring soil quality changes [7,8]. Recently researchers have paid more and more attention to the diversity of soil microbes and their function in agricultural ecology [9–13].

In China, spring crops, such as maize, sorghum and millet, are usually planted from April through May every spring, and harvested in fall. They are the major crops in the Loess Plateau area of China. Wheat is also a very common crop, grown on the North China Plain and in some northern provinces. Wheat is traditionally planted in fall (winter wheat) or spring (spring wheat) and harvested in summer year by year. The disadvantages of this continuous cropping system have been confirmed to reduce microbial diversity significantly, decreasing the fertility of the soil after wheat harvest. This happens not only in wheat, but also in peanut (*Arachis hypogaea*), soybean (*Glycine max*), maize (*Zea mays*), and black pepper (*Piper nigrum* L.) [14–21]. As a practice of growing two or more crops in close proximity during the same growing season, intercropping is widely used in traditional Chinese agricultural production. Intercropping can improve multiple cropping index and soil quality, reduce fertilizers input, maximize the effective use of limited resources, and maintain high grain yields compared with the sole-cropping system [22–25]. Crop productivity increases under intercropping systems were due not only to an enhanced nutrition uptake and light capture, but also to other mechanisms, such as phosphorus mobilization in the rhizosphere [26]. In addition, soil microbial diversity could be affected by different agricultural practices, such as alternative systems, land-use change, fertilization and row ratio of the intercrops [6,27–29]. Moreover, other studies have revealed the close relationship between aboveground plant diversity and underground microbial diversity [30–32]. Thus far, rhizosphere microbial diversities have been studied in several crops including peanut, wheat, maize, soybean, cucumber, onion, and garlic [29,33]. However, very few reports have been published about rhizosphere soils’ microbial diversities when different types of spring crops are sown with a different cropping system in wheat fields after harvest. In our study, we chose ten common spring crops in northern China which are variable in root and shoot systems, including hulled oat (*Avena sativa*), mung bean (*Vigna radiate*, Zhonglv-1), foxtail millet (*Setaria italica*, Jinggu-6), barley (*Hordeum vulgare*, peasant variety), rape (*Brassica campestris*, Wuyueman), sunflower (*Helianthus annuus*, Jinkui-6), carrot (*Daucus carota* var. *Sativus*, Sanhongqicunseng), flax (*Linum usitatissimum*, Jinya-7), naked Oat (*Avena nuda*, peasant variety) and tobacco (*Nicotiana tabacum*, G-80). The rhizosphere microbial diversity was hypothesized to be significantly different depending on different crops as well as different planting systems. To test our hypotheses, we collected data from the above 10 sole-cropped samples as well as the following four intercropping systems: buckwheat (*Fagopyrum esculentum* Moench)-peanut (*Arachis hypogaea*), glutinous millet (*Panicum miliaceum*)-peanut, peanut-sorghum (*Sorghum bicolor* Moench) and peanut- foxtail millet. Then, we sequenced the 16S rDNA fragments in order to examine the microbial species, so as to determine detailed microbial community changes among rhizosphere soils.

## Material and Methods

### Field site description

Two field experiments were conducted in the Experimental Farm of Shanxi Agricultural University at Taigu County, Shanxi Province. The fields are located at 37°25' N and 112°35' E. The fields were left fallow from summer to winter after the winter wheat was harvested on June 28, 2011. Soil samples of cultivated horizon (20 cm) contained total nitrogen of 1.80 g kg^-1^, organic matter of 12.6 g kg^-1^, available N 53.6 mg kg^-1^, available P 9.6 mg kg^-1^ and exchangeable K 137.5 mg kg^-1^. They were measured by using semi-micro Kjeldahl, potassium dichromate volumetry, alkaline hydrolysis diffusion method, NaHCO_3_ extract-colorimetric method, and ammonium acetate flame photometric method, respectively.

### Experimental design

To investigate bacterial community diversity in the rhizosphere soil of crops planted in the wheat-planted field, PCR-DGGE (denaturing gradient gel electrophoresis) of the V3 region of 16S ribosomal DNA (rDNA) was performed. The crops selected for this study included ten spring crops from the sole-cropping experiment and four from the intercropping experiment (Table 1). All experiments were conducted in triplicate.

**Table 1:** Parameters for crop cultivation in sole cropping and intercropping experiments.

Experiment	Crops	Row spacing (cm)	Hole spacing (cm)	Planting depth (cm)	Plants per hole (cm)
**Sole-cropping**	Hulled oat	30	10	5	3
	Mung bean	40	30	4	3
	Foxtail millet	30	6	4	3
	Barley	30	10	5	3
	Rape	15	10	5	1
	Sunflower	80	40	10	1
	Carrot*	30	15	-	1
	Flax	15	10	3	3
	Naked oat	30	10	5	3
	Tobacco*	40	40	-	1
**Intercropping**	The main crop-Peanut	40	15	5	2
	/Buckwheat	-	3	3	1
	/Glutinous millet	-	6	4	3
	/Sorghum	-	20	5	1
	/ Foxtail millet	-	6	4	3
	CK1-Single peanut	40	15	5	2
	CK2-Single buckwheat	30	3	3	1
	CK3-Single glutinous millet	30	20	5	3
	CK4-Single sorghum	30	6	4	1

*Above the mentioned crops were directly sown to soil with the exception of tobacco and carrot seedlings, which were transplanted (row spacing and plant spacing) to the field after establishment in seedling-beds for 25 days.

*Sole-cropping Experiment*: Ten spring crops were planted by hole sowing in the field on April 27, 2012, including hulled oat (*Avena sativa*, the name of variety is MANOTICK), naked Oat (*Avena nuda*, peasant variety), barley (*Hordeum vulgare*, peasant variety), foxtail millet (*Setaria italica*, Jinggu-6), mung bean (*Vigna radiate*, Zhonglv-1), flax (*Linum usitatissimum*, Jinya-7), rape (*Brassica campestris*, Wuyueman), carrot (*Daucus carota* var. *sativus*, Sanhongqicunseng), sunflower (*Helianthus annuus*, Jinkui-6), and tobacco (*Nicotiana tabacum*, G-80) (Table 1). They were selected because they are main spring crops in northern China.

**Fig 1:**
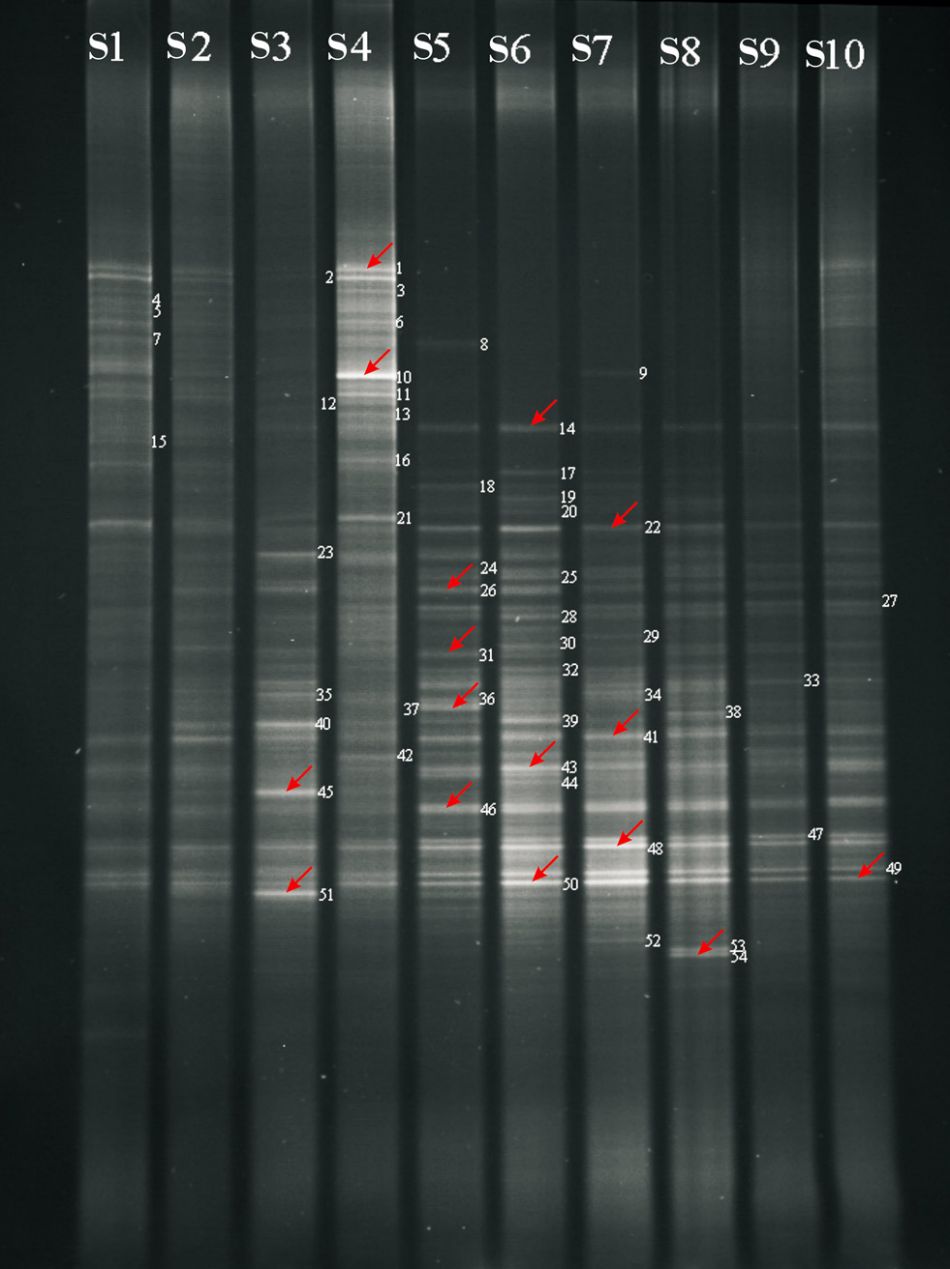
DGGE analysis of bacterial community profiles of 16S rDNA PCR amplification products of rhizosphere soil samples S1-S10 in intercropping experiment. Soil samples were labeled as in Table 2. S1, CK1-Single Peanut, S2, CK2-Single Buckwheat, S3, CK3-Single Sorghum, S4, CK4-Single Glutinous millet, S5, Intercropping Buckwheat (/Peanut), S6, Intercropping Peanut (/Buckwheat), S7, Intercropping Glutinous millet (/Peanut), S8, Intercropping Peanut(/Glutinous millet), S9, Intercropping Peanut(/Foxtail millet), S10, Intercropping Sorghum(/Peanut). Arrows indicate the selected bands for further DNA sequencing in some soils. The numbers without arrows show the visible bands under UVI Bioimaging system without further DNA sequencing.

*Intercropping Experiment*: Crops selected for the intercropping experiment mainly depended on their variations in root and shoot systems. Peanut (*Arachis hypogaea*) was chosen as the main crop, and buckwheat (*Fagopyrum esculentum* Moench), glutinous millet (*Panicum miliaceum*), foxtail millet (*Setaria italica*), and sorghum (*Sorghum bicolor* Moench) were planted as intercrops in the field on April 27, 2012. In addition, buckwheat, peanut, glutinous millet, foxtail millet, and sorghum were also planted singly as the control (CK) (Table 1**)**. However, due to the unsuccessful PCR, foxtail millet was not used in the control group in DGGE analysis (Fig 1).

In both experiments, the row spacing, hole spacing, planting depth and plants per hole of each crop were summarized in Table 1. Each crop was planted 6 rows and the length per row was 2 m. All crops were conducted in triplicate, and named as A, B, and C groups in the sole cropping experiment and D, E, and F groups in both experiments, respectively.

### Soil sampling

For each crop, soil samples were collected on three different sampling sites (A, B and C; or D, E, and F) at the flowering stage. An 8-cm diameter soil auger was used to drill into the ground around the crop roots, and the soil samples were obtained from 0–20 cm layer. soil samples were placed into sterile petri plates and roots were taken out. Non-rhizoshpere soil were removed by shaking the roots gently, soils remaining on the roots were collected as rhizoshpere soil. Three random sampling points were chosen for each sampling site (such as A) with a minimum distance of 2 m between individual sampling points. Nine random single samples of rhizosphere soil were collected and thoroughly mixed in order to obtain a composite sample. The composite samples representing each crop were sieved by a 2-mm sieve, collected in sealed individual bags, and stored at -20°C prior to DNA extraction(Table 2).

**Table 2:** Code number for 20 collected soil samples.

**Intercropping Experiment**	S1	S2	S3	S4	S5
	Single Peanut	Single Buckwheat	Single Sorghum	Single Glutinous millet	Intercropping Buckwheat (/Peanut)
	S6	S7	S8	S9	S10
	Intercropping Peanut (/Buckwheat)	Intercropping Glutinous Millet (/Peanut)	Intercropping Peanut (/Glutinous millet)	Intercropping Peanut (/Foxtail millet)	Intercropping Sorghum (/Peanut)
**Sole-cropping Experiment**	S11	S12	S13	S14	S15
	Hulled oat	Mung bean	Foxtail millet	Barley	Rape
	S16	S17	S18	S19	S20
	Sunflower	Carrot	Flax	Naked oat	Tobacco

### PCR-DGGE bacterial community analysis

Species of the complex bacterial community were assayed by a genotypic fingerprinting approach using the PCR-DGGE technique. Total DNA was extracted directly from the soil by using a bead-beating method (SoilGen DNA Kit, Enterprise Group CWBIO Co., Ltd., Beijing, P. R. China).

#### PCR amplification of 16S rDNA V3 hypervariable region

Target part of the 16S rDNA V3 hypervariable region (230 bp) was amplified in 50 μL of reaction mixture using universal bacterial specific primers *F*357-GC (5'-CGCCCGCCGCGCCCCGCGCCCGGCCCGCCGCCCCCGCCCCCCTACGGGAGGCAGCAG-3') and *R*518(5'- ATT ACC GCG GCT GCT GG -3'). A 230-bp PCR product was obtained. The reaction mixture compositions were 41.25 μL of ddH_2_O, 5 μL of 10×reaction buffer (including 2.0 mM MgCl_2_), 1 μL of dNTP (10 mM), 1 μL of *F*357-GC (10 μM), 1 μL of *R*518 (10 μM), 0.25 μL of Taq enzyme (5 U/μL), and 0.5 μL of DNA template, respectively. DNA samples were amplified PCR on a T3000 Thermocycler (Gene Amplification PCR System, BBI, Canada) using the following thermal cycling scheme: initial denaturation at 94°C for 4 min, 30 cycles of denaturation at 94°C for 30 s, annealing at 56°C for 1 min, and extension at 72°C for 30 s, followed by a final extension period at 72°C for 7 min. An aliquot of 3 μl of the PCR product was checked by agarose gel electrophoresis (1.5%, w/v, agarose; 5%, w/v, Goldview dye liquor; 1×TAE buffer, 120 V, 30 min) (DYY-8 Stable Voltage and Steady Flow Electrophoresis Apparatus, Qite Analytical Instrument Co., Ltd., Shanghai, P. R. China). Gels were visualized and digitized with UVI Gene Genius Bioimaging System (Gene Genius, USA).

#### DGGE analysis

**Fig 2:**
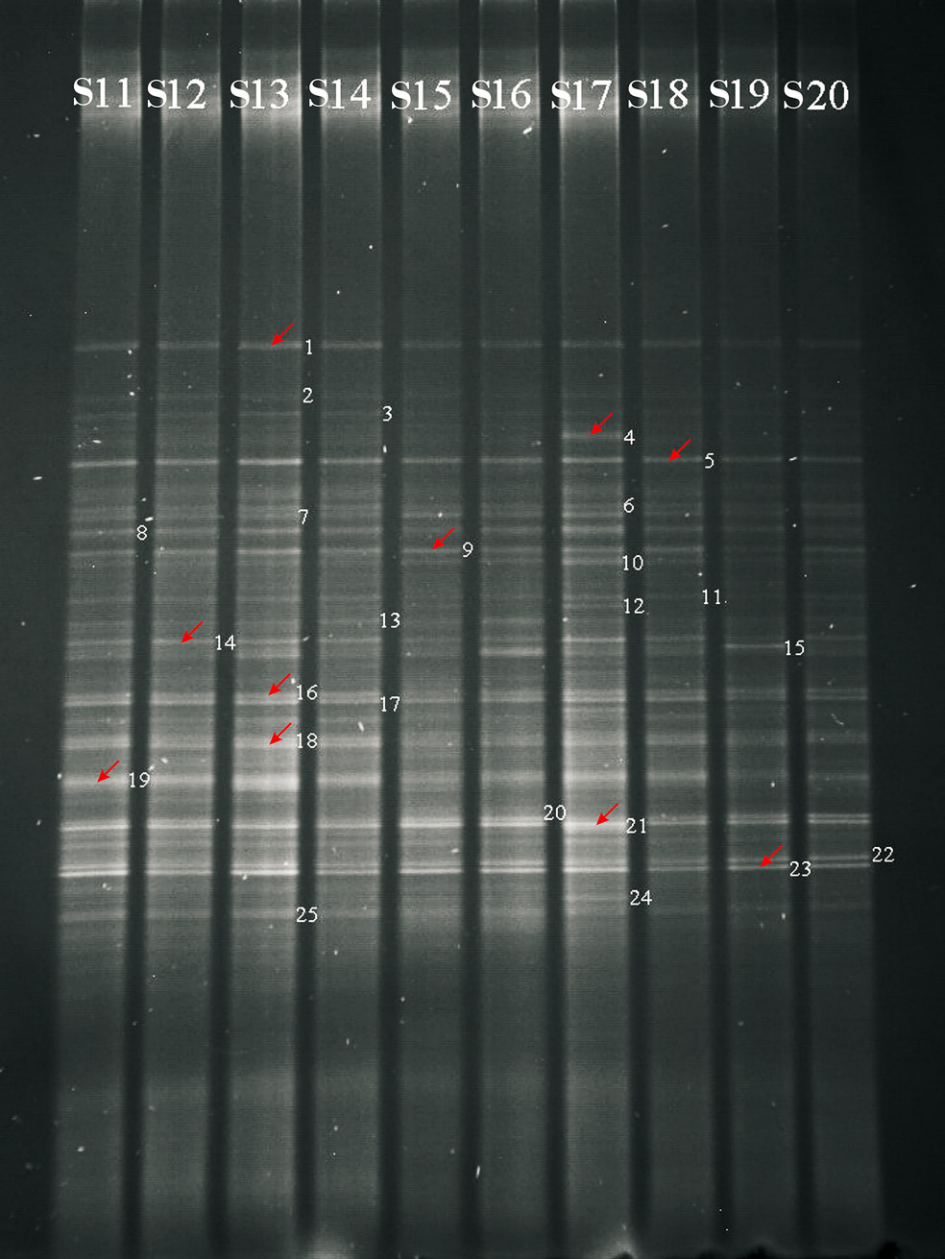
DGGE analysis of bacterial community profiles of 16S rDNA PCR amplification products of rhizosphere soil samples S11-S20 in sole-cropping experiment. Soil samples were labeled as Table 2. S11, Hulled oat, S12, Mung bean, S13, Foxtail millet, S14, Barley, S15, Rape, S16, Sunflower, S17, Carrot, S18, Flax, S19, Naked oat, S20, Tobacco. Arrows indicate the selected bands occurring either in all soils or in some soils only. The numbers without arrows show the visible bands under UVI Bioimaging system without further DNA sequencing.

The DGGE of PCR products (400 ng) was carried out by using a D-Code Mutation Detection System instrument (Bio-Rad, USA). Gels were prepared and run in the following conditions: 8% (w/v) polyacrylamide (acrylamide/bis-acrylamide = 37.5:1), 1×TAE buffer, linear gradient from 30% to 60% denaturant (where 100% denaturing acrylamide was defined as containing 7 mol/L urea and 40% v/v formamide), 4.0 h at 180 V and 60°C. Following this process, gels were washed in ultrapure water, loaded in dye liquor with 5% Goldview, shaken for 30 min (Shaking Tables, P. R. China), and then visualized by the UVI Gene Genius Bioimaging System (Figs 1 and 2).

In each lane of the DGGE gel, the visualization of DNA bands could respond to relative bacteria biomass, and the number of electrophoresis bands could directly reflect the genetic diversity of bacterial community in rhizosphere soil samples. These individual bands represented different gene fragments or different stable structural conformations of bacterial 16S rDNA V3 hypervariable region. The higher the band number is, the more diverse the bacterial community is.

For unknown reasons, we only get successful PCR products from the 10 soil samples in the intercropping experiment: CK1-Single Peanut (S1 in Fig 1), CK2-Single Buckwheat (S2), CK3-Single Sorghum (S3), CK4-Single Glutinous millet (S4), Intercropping Buckwheat (/Peanut) (S5), Intercropping Peanut (/Buckwheat) (S6), Intercropping Glutinous millet (/Peanut) (S7), Intercropping Peanut(/Glutinous millet) (S8), Intercropping Peanut(/Foxtail millet) (S9), Intercropping Sorghum(/Peanut) (S10). S1-S10 are shown in Fig 1. Although CK-foxtail millet, Intercropping Peanut (/Sorghum) and Intercropping Foxtail millet (/Peanut) are not included in further DGGE analysis, they do not affect the final conclusion since the horizontal comparison between S9 and CK1 and between S10 and CK3 can also represent the bacterial community change in certain levels.

#### DGGE gel bands recovery

The specific bands were identified by direct sequencing of DGGE bands (bands indicated by arrows in Figs 1 and 2). The selected bands were either general (occurring in all soils) (bands No. 1, 5, 9, 21, and 23 in Fig 2), or specific (occurring in some soils only) (bands No. 1, 10, 14, 22, 26, 31, 37, 41, 43, 45, 46, 48, 49, 50, 51, and 54 in Fig 1; bands No. 4, 14, 16, 18 and 19 in Fig 2). These selected bands were cut fully from the DGGE gel and put in a 1.5 ml centrifuge tube. DNA was re-isolated by the SK1135 UNIQ-10 Column Kit (Shanghai Sangon Biological Engineering Technology & Service Co., Ltd., P. R. China), and re-amplification targeting DGGE gel bands recovery was achieved with the following primers: F357 5'- CC TAC GGG AGG CAG CAG -3', R518 5'- ATT ACC GCG GCT GCT GG -3'. Expected amplification fragment size was about 170 bp. The PCR mixture compositions and reaction conditions were the same as above (2.4.1). The PCR products were recovered by the SK1131 UNIQ-10 Column Kit. All reactions were performed in triplicate.

### Cloning and sequencing

Target fragments were TA ligated to Takara pMD^®^18-T vector. The ligation products were transformed into *E*. *coli* JM109 Competent Cells made by CaCl_2_ method. Transformants were selected on LB agar plates supplemented with ampicillin (100 mg/L), X-gal (20 mg/mL) and IPTG (isopropyl-beta-Dthiogalactopyranoside) (100 mmol/L) following standard methods. White colonies (transformants) were picked randomly from the plates for colony PCR. Plasmid DNA carrying inserts were extracted by an SK1191 UNIQ-10 Column Extraction Kit and sequenced with M13+/- primers. DNA sequences were manually checked and edited where necessary using Chromas Lite version 2.01 software (Technelysium Pty Ltd, Australia). These partial 16S rDNA sequences were compared with sequences deposited in the GenBank database using Blast (http://blast.ncbi.nlm.nih.gov/). The new DNA sequences had been submitted to GenBank with accession numbers KP229386- KP229411.

### Analysis of DGGE patterns

A diversity index is a mathematical measurement of species diversity in a community. The Shannon diversity index (*H*) and Simpson's (*D*) index have been commonly used to characterize species diversity in a community [34]. DNA bands in gels were identified by Quantity One software (Bio-Rad, USA). Both Shannon diversity index (*H*) and Simpson's index (*D*) were calculated based on the number and relative intensities of DGGE bands. In detail, Shannon's (*H*) and Simpson's (*D*) indices were calculated as follows, respectively: *H* = −∑(*Pi*)(ln *Pi*), $D=1-\sum P_{i}^{2}$, where *pi* is the proportion of *i*th phylotype. To further understand the differentiation among DNA fragments from rhizosphere soil samples, the clustering algorithm was used to calculate the dendrograms of DGGE band profiles via an unweighted pair group method with arithmetic means (UPGMA). Statistical analyses were performed in ANOVA program of SAS9.1.3 software. A probability value (*P*) of <0.05 was considered statistically significant.

## Results

### PCR–DGGE analysis of soil samples

To investigate the bacterial diversity level, we used the cluster analysis generated by a UPGMA dendrogram to calculate the diversity indices. As seen from Fig 1 and Table 3, DGGE profiles of PCR products are different in terms of the location, visualization (intensities), and numbers of bands from soil samples S1-S10 in the intercropping experiment. The same was true in Fig 2 and Table 4 for soil samples S11-S20 in the sole-cropping experiment. Some strong and rather characteristic bands were observed in DGGE patterns under intercropping systems, which were absent under monoculture (sole-crops). For example, intercropping buckwheat soil increased such bands shown as code number 14, 22, 26, 37 and 46 (S5 in Fig 1), while single buckwheat had no such bands (S2 in Fig 1) The same situation occurred in intercropping glutinous millet, peanut and sorghum. Intercropping glutinous millet soil of S7 increased bands 14, 22 and 41 compared to the single glutinous millet soil of S4; Intercropping peanut soil of S6 increased bands 14, 26, 43 and 50 compared to the single peanut soil of S1. Intercropping sorghum soil of S10 was found to have bands 46 and 49 compared to the single sorghum soil of S3 (Fig 1).

**Table 3:** Simpson's and Shannon's method/ Log base of rhizosphere soil samples S1-S10 in intercropping experiment.

Soil samples	Simpson's Index	Shannon's Index	Number of Species
S1	0.952	3.045	21
S2	0.941	2.833	17
S3	0.947	2.944	19
S4	0.960	3.219	25
S5	0.968	3.434	31
S6	0.971	3.555	35
S7	0.963	3.296	27
S8	0.968	3.434	31
S9	0.950	2.996	20
S10	0.969	3.466	32

Soil samples were labeled as Table 2. S1, CK1-Single peanut, S2, CK2-Single buckwheat, S3, CK3-Single sorghum, S4,CK4-Single glutinous millet, S5, Intercropping buckwheat(/peanut), S6, Intercropping peanut(/buckwheat), S7, Intercropping glutinous millet(/peanut), S8, Intercropping peanut(/glutinous millet), S9, Intercropping peanut(/foxtail millet), S10, Intercropping sorghum(/peanut). Indices of all soil samples from two cropping systems were compared by using Duncan's multiple comparison. Significant differences were observed among all soil samples (*P*<0.05).

**Table 4:** Simpson's and Shannon's method/ Log base of rhizosphere soil samples S11-S20 in sole-cropping experiment.

Soil samples	Simpson's index	Shannon's index	Number of species
S11	0.962	3.258	26
S12	0.962	3.258	26
S13	0.960	3.219	25
S14	0.955	3.091	22
S15	0.938	2.773	16
S16	0.955	3.091	22
S17	0.963	3.296	27
S18	0.958	3.178	24
S19	0.947	2.944	19
S20	0.944	2.890	18

Soil samples were labeled as Table 2. S11, Hulled oat, S12, Mung bean, S13, Foxtail millet, S14, Barley, S15, Rape, S16, Sunflower, S17, Carrot, S18, Flax, S19, Naked oat, S20, Tobacco. Indices from different samples in sole cropping system were compared by using Duncan's multiple comparison. Significant differences were observed among all soil samples (*P*<0.05).

In general, band numbers from intercropping soil samples were higher than those from single crop soil samples, with the exception of peanut/foxtail millet intercropping. The highest number was 35 from the peanut soil sample intercropped with buckwheat (S6 in Fig 1). Under peanut-foxtail millet intercropping systems, there are only 20 visible bands found in rhizosphere soil samples, which is the least in DGGE profiles (S9 of Fig 1). There were different band numbers between 16 and 27 among ten crops, 27 for carrot and 16 for rape in S17 and S15 respectively, under sole-cropping (Fig 2). The results revealed that the compositions of rhizosphere soil bacterial communities were different among cropping systems and crop species.

In addition, we found common rhizosphere bacteria in two cropping systems. In the sole-cropping system, uncultured *Acidobacteria* bacterium (band code 1 in Fig 2), *Persicobacter psychrovividus* (code 5 in Fig 2), uncultured *Sphingobacteriales* bacterium(code 9 in Fig 2), *Pseudomonas fulva*(code 21 in Fig 2), and uncultured bacterium(code 23 in Fig 2) are commonly found across all crops.

In the intercropping system, uncultured *Acidobacteria* bacterium code (code14 in Fig 1), uncultured *Gemmatimonadetes* bacterium (code 22 in Fig 1), uncultured *Chloroflexi* bacterium (code 46 in Fig 1), *Pseudomonas libanensis* (code 48 in Fig 1), uncultured bacterium (code 49 in Fig 1), and uncultured *delta proteobacterium* (code 50 in Fig 1) are found in all intercropped samples. The common bacteria indicated that there were some shared characteristics between each cropping system respectively.

### Genetic diversity of rhizosphere bacterial communities in intercropping soil samples S1-S10

**Fig 3:**
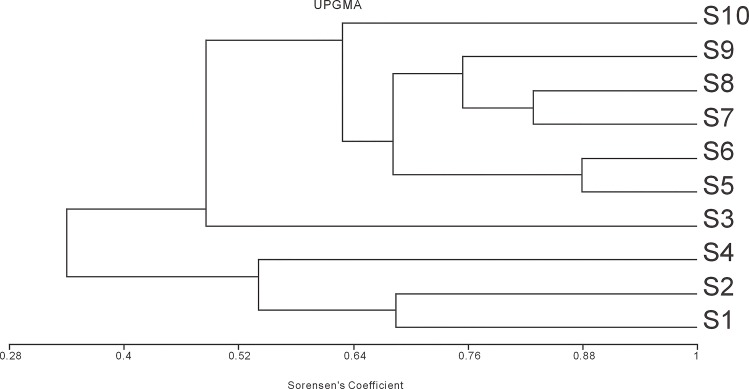
UPGMA dendrogram constructed with DGGE profiles of bacterial 16S rDNA PCR amplification products from rhizosphere soil samples S1-S10. Soil samples were labeled as Table 2. S1, CK1-Single peanut, S2, CK2-Single buckwheat, S3, CK3-Single sorghum, S4,CK4-Single glutinous millet, S5, Intercropping buckwheat(/peanut), S6, Intercropping peanut(/buckwheat), S7, Intercropping glutinous millet(/peanut), S8, Intercropping peanut(/glutinous millet), S9, Intercropping peanut(/foxtail millet), S10, Intercropping sorghum(/peanut).

As seen from Table 3, diversity indices of the soil bacterial community were significantly higher under intercropping (S5, S7 and S10) than under monoculture (S2, S4 and S3) with the exception of the peanut/ foxtail millet system (S9) (*P*<0.05). These findings are basically consistent with the increases in band number and intensity observed in the DGGE profiles (Fig 1). Fewer bands were found under monoculture, which indicated that less diverse bacterial communities were present in soil under monoculture. Among six intercropping soil samples experimented, peanut soil sample intercropped with buckwheat had the highest diversity indices of the soil bacterial community. To further understand the bacterial community structures of each sample, we built a UPGMA clustering tree by using the 16S rDNA fragment (about 230 bp) and community Sorensen's coefficient, from sample S1-S10 (Fig 3). The clustering analysis (Table 3, Fig 3) indicated that the community structures of bacteria in intercrop soil samples could not be reverted back to the conditions found in monoculture crops, even though the diversity indices of the bacterial community in intercrop soil samples (S7 and S8) were similar to those of monoculture crops soil samples (S4 and S1). For intercropping rhizosphere soil samples, two main clusters could be detected in the condition of Sorensen's Coefficient with 0.4 (Fig 3). One cluster was found in S4 (single glutinous millet) as well as S1 and S2 (single peanut and buckwheat). The other cluster was observed in S3 (single sorghum) and all intercrop soil samples (S5、S6、S7、S8、S9、S10). Therefore, the changes in bacterial community structure were most distinct in intercropping systems. The intercropping system altered bacterial community structures in the rhizosphere as opposed to that in monocultured crops. So, intercropping buckwheat, glutinous millet and sorghum with peanut could significantly increase microbial community diversity compared to sole-cropping these crops in the fields after wheat harvest (*P*<0.05).

### Genetic diversity of rhizosphere bacterial communities in sole-cropping soil samples S11-S20

**Fig 4:**
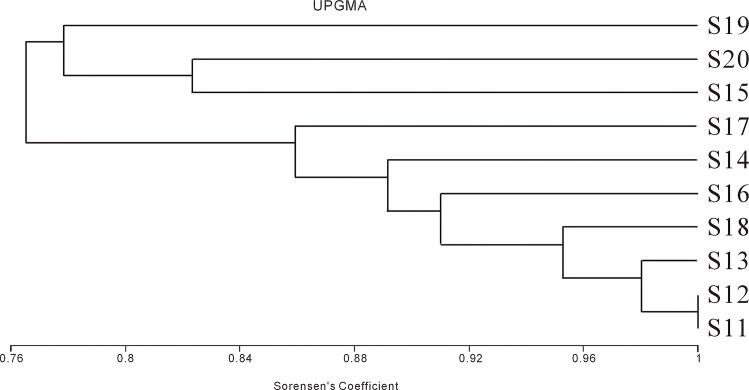
UPGMA dendrogram constructed with DGGE profiles of bacterial 16S rDNA PCR amplification products from rhizosphere soil samples S11-S20. Soil samples were labeled as Table 2. S11, Hulled oat, S12, Mung bean, S13, Foxtail millet, S14, Barley, S15, Rape, S16, Sunflower, S17, Carrot, S18, Flax, S19, Naked oat, S20, Tobacco.

As seen from Table 4, the diversity indices of these ten sole-cropping soil samples were significantly different (*P*<0.05), consistent with the increases in band number and intensity observed in the DGGE profiles (Fig 2). These findings revealed that the diversity of rhizosphere soil bacterial communities was significantly affected by different kinds of crops (*P*<0.05). Among these different crops, carrots had the highest diversity index (S17 of Table 4). Similarly, to understand the bacterial community structures, a UPGMA clustering tree was built by using the16S rDNA sequence from sample S11-S20 (Fig 4). Even though the diversity indices of bacterial communities in the rhizosphere soil samples of different crops were similar to each other in Table 4, the population structures of bacteria in these soil samples had differences as indicated in Fig 4. Under sole-cropping, three main clusters are calculated to have 80% similarity (Fig 4). Ten crops were clustered into 9 classes when the similar value was 1. So, in terms of bacterial community diversity, sole cropping carrots (Sample S17) was better than sole cropping hulled oats (S11) and mung beans (S12) in the fields after wheat harvest. The worst choice was sole-cropping rape (S15).

### Microbial community changes in rhizosphere soil samples

After sequencing of the DNA products cut from the gel bands in Figs 1 and 2, the obtained sequences were further analyzed by BLASTN on the NCBI webserver. Tables 5 and 6 summarized the results of comparing sequences for the excised bands with reference strains from Genbank. 26 excised bands were selected from 14 rhizosphere soil samples of different crops. In total, 26 bacterial species were identified, each band representing a different bacterial species. The results indicated that the numbers of uncultured rhizosphere soil bacterium increased under intercropping systems and that some bacteria were present only in several crops’ monoculture, such as *Gemmatimonas aurantiaca* and *Flavobacterium* sp. present in glutinous millet (*Panicum miliaceum*) (S4), while uncultured soil bacterium and *Pseudomonas fulva* was present in sorghum (*S*. *bicolor* Moench) (S3). Under sole-cropping of ten crops, there were also changes in microbial communities of rhizosphere soil. For example, *Empedobacter brevis* was present only in the rhizosphere soil-grown carrot. Uncultured soil bacterium, uncultured compost bacterium and *Bacillus vallismortis* was absent in the grown rape. With the identifications of bacterial species, our study demonstrated that cropping systems and crop species had obvious effects on bacteria community diversity in the rhizosphere soils.

**Table 5:** Identification of bacteria for sixteen bands sequenced in the intercropping experiment.

Code number of bands on Fig 1	Accession numberin NCBI	V3 Region	Bacterium identification
1	KP229386	186bp	*Gemmatimonas aurantiaca*
10	KP229387	189bp	*Flavobacterium* sp.
14	KP229388	169bp	uncultured *Acidobacteria* bacterium
22	KP229389	194bp	uncultured *Gemmatimonadetes* bacterium
26	KP229390	169bp	uncultured bacterium
31	KP229391	187bp	uncultured *Flavisolibacter* sp.
37	KP229392	194bp	*Luteibacter rhizovicinus*
41	KP229393	192bp	*Acidovorax* sp. 'smarlab133815'
43	KP229394	169bp	*Asticcacaulis excentricus*
45	KP229395	190bp	uncultured soil bacterium
46	KP229396	170bp	uncultured *Chloroflexi* bacterium
48	KP229397	194bp	*Pseudomonas libanensis*
49	KP229398	170bp	uncultured bacterium
50	KP229399	193bp	uncultured *delta proteobacterium*
51	KP229400	194bp	*Pseudomonas fulva*
54	KP229401	171bp	uncultured bacterium

**Table 6:** Identification of bacteria for ten bands sequenced in the sole-cropping experiment.

Code number of bands on Fig 2	Accession number in NCBI	V3 Region	Bacterium identification
1	KP229402	191bp	uncultured *Acidobacteria bacterium*
4	KP229403	189bp	*Empedobacter brevis*
5	KP229404	189bp	*Persicobacter psychrovividus*
9	KP229405	188bp	uncultured *Sphingobacteriales bacterium*
14	KP229406	195bp	*Geobacter* sp.
16	KP229407	170bp	uncultured soil bacterium
18	KP229408	174bp	uncultured compost bacterium
19	KP229409	195bp	*Bacillus vallismortis*
21	KP229410	194bp	*Pseudomonas fulva*
23	KP229411	194bp	uncultured bacterium

## Discussion

Previous studies have demonstrated that the bacterial diversity in rhizospheres could be influenced by many factors, such as soil type, nutrition, management practice, soil properties, varietal differences within a species, plant age, plant species and plant genotype [35–39]. However, the main factor affecting soil microbiological characteristics was related to different types of land use, for example, organic farming and long-term fertilizer management, as well as soil conditions including nitrate concentrations, organic matters etc. [38,40–43]. It has also been demonstrated that the soil conditions—for example, nitrate concentration—are correlated with soil microbes, like nitrogen-fixing bacteria and nitrifuing bacteria [44,45]. Furthermore, cropping systems could affect soil microbial communities, and the abundance and community structure of soil bacterial groups changed in response to management practice changes [6,46–47]. Mixed cropping promoted significant increase of rhizosphere microbial population, and increased enzyme activity and soil nutrition in the immature loess subsoil when compared with monoculture [48–50]. In our study, the community structures of rhizosphere soil bacteria (Figs 1 and 2) and the diversity indices (Tables 3 and 4) differed among spring crops in wheat-planted fields, indicating that rhizosphere soil bacterial populations significantly vary as a response to both different cropping systems and different crop species. This conclusion is consistent with previous research. Futhermore, intercropping peanut with buckwheat, sorghum or glutinous millet was beneficial to the composition of bacterial communities in rhizosphere soils. In the control group of single glutinous millet (S4, Fig 1), we found more visible bands than others. This was not in agreement with not only the previous results [33,51] but also the results of other intercropping systems in the present study. Under peanut-foxtail millet intercropping systems (S9, Fig 1), are the least visible bands for bacterial community in DGGE profiles. Considering that the composition of bacterial communities in soil can be affected either directly by changing the host plant physiology or indirectly by changing the patterns of root exudation [52,53], root exudates are crucial determinants of rhizosphere microorganism diversity [54–56]. Thus, one possible explanation for this inconsistency might be because microbe and root exudates change their interactions. However, the exact mechanism of foxtail millet-peanut, foxtail millet-microbes, glutinous milliet-microbes and microbe-microbe interactions remains to be further explored.

Rhizosphere soil is influenced by plant roots, which are selected for specifically adapted microbial communities [57,58]. The more prosperous the root is, the more root exudates there are. These materials, made up of saccharides, organic acid, amino acid, phenolic compound, and so on, could provide numerous nutritional energy materials and make ecological distribution change [59]. The amount and kind of root exudates and allelopathy differ between crop species, and the differences could stimulate species-specific shifts in the soil microbial community [38,60–61]. Moreover, plants can exert a highly effective effect on the soil bacterial community that is at least as great as that of the soil [40]. For example, organic acid of in root exudates of three hydroponic plants (*chlorophytum comosum*, *ipomoea aquatica*, *oenanthe javanica*) has a promotion effect on ammonifying bacteria and denitrifying bacteria, but exhibits inhibition effect on nitrobacteria and nitrosobacteria [62]. Our study also demonstrated the obvious effect of different kinds of plants on rhizosphere bacterial communities with shifts in the composition of dominant populations through DGGE profiles. Carrot had the most visible bands for bacterial community (27 bands), while rape had the least visible bands in bacterial profiles (16 bands, Fig 2, Table 4). Diversity indices were consistent with the DGGE profile (Fig 2).

Bacterial species recognition by sequencing 16S rDNA fragments showed that crop species and intercropping systems could stimulate specific bacterial species, while also causing the loss of others, thereby influencing microbial communities of rhizosphere soils. Our study observed a significant stimulation of uncultured *Acidobacteria* bacterium, uncultured *Gemmatimonadetes* bacterium, and *Acidovorax* sp. 'smarlab133815' in intercropping glutinous millet and the loss of *Gemmatimonas aurantiaca* and *Flavobacterium* sp. in intercropping glutinous millet. Furthermore, the coexistence of plant species increased bacterial diversity because of the close association between the composition of the soil microbial community and the plant diversity [63]. In the intercropping experiment, various dominant and functional bacteria were enhanced (uncultured *Flavisolibacter* sp., *Luteibacter rhizovicinus*, uncultured *Chloroflexi* bacterium, uncultured *Acidobacteria* bacterium and uncultured *delta proteobacterium* under peanut/buckwheat intercropping, and uncultured *Gemmatimonadetes* bacterium, *Pseudomonas libanensis* and uncultured bacterium under peanut/glutinous millet intercropping), which implies a potential relationship between the yield increase of intercropping plants and microbial diversity when compared with sole-cropping [64]. This implication had also been confirmed in the intercropping systems of maize, alfalfa—Siberian wild rype, buckwheat, millet and sorghum with peanut [65, 66]. However, the effects of these bacteria on specific crop productivity still need to be further studied.

In addition, our study suggested a relatively strong species-specific pattern on bacterial community structure in rhizosphere soil. In the sole-cropping experiment, the bacterial community diversity in carrots (Sample S17) was observed to be higher than hulled oats (Sample S11) and mung beans (Sample S12). The lowest was found in rape (Sample S15). Futhermore, *Empedobacter brevis* was only found in the rhizosphere soil grown carrots, while uncultured soil bacterium, uncultured compost bacterium and *Bacillus vallismortis* were all absent in those grown with rape. More interestingly, however, *Empedobacter brevis* is a typically pathogenic bacterium to plants, so carrot is suggested to be prudently used in wheat fields after harvest. Further studies should be conducted to examine the detailed mechanisms of these uncultured rhizosphere soil bacteria and their interactions with specific crop species.

## Conclusions

Our study highlighted that variation of bacterial community diversity in rhizosphere soil is related with different crops as well as different planting systems. Intercropping systems could induce an increase of some functional and uncultured rhizosphere soil bacteria, suggesting a potential relationship between yield increase of intercropping crops and microbes when compared with sole-crops. Intercropping buckwheat, glutinous millet and sorghum with peanut could increase microbial community diversity when compared with their sole-cropping systems, with several specific bacteria found to be promoted in intercropping peanut/buckwheat and peanut/glutinous millet. Bacterial community diversity is more species dependent in sole-crops. Besides, *Empedobacter brevis*, a typical plant pathogen, was only found in the carrot rhizosphere, suggesting that carrot should be prudently used in wheat fields after harvest. Our study strongly suggested that sorghum, glutinous millet and buckwheat could be considered for intercropping crops with peanut; and hulled oat, mung bean or foxtail millet could be considered to be planted in wheat fields after harvest in north China. Further studies should be conducted in order to examine the exact mechanisms of these uncultured rhizosphere bacteria and their interactions with specific crop species.

## References

[1] Sainia VK, Bhandarib SC, Tarafdar JC. Comparison of crop yield, soil microbial C, N and P, N-fixation, nodulation and mycorrhizal infection in inoculated and non-inoculated sorghum and chickpea crops. Field Crops Res. 2004; 89: 39–47.

[2] Gomes NC, Fagbola O, Costa R, Rumjanek NG, Buchner A, Mendona-Hagler L, et al Dynamics of fungal communities in bulk and maize rhizosphere soil in the tropics. Appl. Environ. Microbiol. 2003; 69: 3758–3766. 12839741 10.1128/AEM.69.7.3758-3766.2003 PMC165189

[3] Doran JW, Zeiss MR. Soil health and sustainability: managing the biotic component of soil quality. Appl. Soil Ecol. 2000; 15: 3–11.

[4] Wakelin SA, Macdonald LM, Rogers SL, Gregg AL, Bolger TP, Baldock JA. Habitat selective factors influencing the structural composition and functional capacity of microbial communities in agricultural soils. Soil Biol. Biochem. 2008; 40: 803–813.

[5] Janvier C, Villeneuve F, Alabouvette C, Edel-Hermann V, Mateille T, Steinberg C. Soil health through soil disease suppression: which strategy from descriptors to indicators. Soil Biol. Biochem. 2007; 39: 1–23.

[6] Acosta-Martínez V, Burow G, Zobeck TM, Allen VG. Soil microbial communities and function in alternative systems to continuous cotton. Soil Sci. Soc. Am. J. 2010; 74: 1181–1192.

[7] Maarit Niemi R, Ilse Heiskanena, Kaisa Wallenius, Kristina Lindström. Extraction and purification of DNA in rhizosphere soil samples for PCR-DGGE analysis of bacterial consortia. J. Microbiol. Methods. 2001; 45: 155–165. 11348673 10.1016/s0167-7012(01)00253-6

[8] Brock TD. The study of microorganisms in situ: progress and problems. Symposium of the Society for General Microbiology.1987; 41: 1–17.

[9] Xl Zhang, Ma L, Gilliam FS, Wang Q, Li Ch. Effects of raised-bed planting for enhanced summer maize yield on rhizosphere soil microbial functional groups and enzyme activity in Henan Province, China. Field Crops Res. 2012; 130: 28–37.

[10] Zabaloya MC, Garland JL, Gomez MA. Assessment of the impact of 2,4-dichlorophenoxyacetic acid (2,4-D) on indigenous herbicide-degrading bacteria and microbial community function in an agricultural soil. Appl. Soil Ecol. 2010; 46: 240–246.

[11] Kelley ST, Dobler S. Comparative analysis of microbial diversity in Longitarsus flea beetles(Coleoptera: Chrysomelidae). Genetica. 2011; 139(5): 541–550. 10.1007/s10709-010-9498-0 20844936

[12] Donn S, Almario J, Muller D, Moënne-Loccoz Y, Gupta VVSR, Kirkegaard JA, et al Rhizosphere microbial communities associated with Rhizoctoniadamage at the field and disease patch scale. Appl. Soil Ecol. 2014; 78: 37–47.

[13] Schmalenberger A, Noll M. Bacterial communities in grassland turfs respond to sulphonate addition while fungal communities remain largely unchanged. Eur. J. Soil Biol. 2014; 6: 12–19.

[14] Sun XS, Feng HS, Wan SB, Zuo XQ. Changes of Main Microbial Strains and Enzymes Activities in Peanut Continuous Cropping Soil and Their Interactions(in chinese). Acta Agronomica Sinica. 2001; 27(5):617–621.

[15] Huang YQ, Han LS, Han M, Xiao YN, Yang JF, Han XR. Influence of continuous cropping years on soil enzyme activities of peanuts(in chinese). Chinese journal of oil crop sciences. 2012; 34(1): 96–100.

[16] Sun L. Effect of Soybean Continuous Cropping on The Rhizosphere Soil Nutrition(in chinese). Chinese Agricultural Science Bulletin. 2008; 24(12): 266–269.

[17] Gu Y, Qiu Q, Wang ZM, Chen XF, Wu CS. Effects of Soybean Continuous Cropping on Microbial and Soil Enzymes in Soybean Rhizosphere(in chinese). Scientia Agricultura Sinica.2012; 5(19):3955–3964.

[18] Xing HQ, Xiao ZW, Yan JZ, Ma JC, Meng Y. Effects of continuous cropping of maize on soil microbes and main soil nutrients(in chinese). Pratacultural Science, 2011; 28(10):1777–1780.

[19] Shi P, Gao Q, Wang SP, Zhang Y. Effects of continuous cropping of corn and fertilization on soil microbial community functional diversity(in chinese). Acta Ecologica Sinica. 2010;30(22): 6173–6182.

[20] Zhang LQ, Hao MD, Zang YF, Li LX. Effects of continuous cropping of wheat and alfalfa on soil enzyme activities and nutrients(in chinese).Chinese Journal of Applied Ecology. 2014; 25(11): 3191–3196. 25898616

[21] Xiong W, Li Z, Liu H, Xue C, Zhang R, Wu H, et al The effect of long-term continuous cropping of black pepper on soil bacterial communities as determined by 454 pyrosequencing. PLOS One. 2015 8 28;10(8):e0136946 10.1371/journal.pone.0136946 [PMC free article][PubMed]. 26317364 PMC4552827

[22] Li L, Sun JH, Zhang FS, Li XL, Yang SC, Rengel Z. Wheat/maize or wheat/soybean strip intercropping: I. Yield advantage and interspecific interactions on nutrients. Field Crops Res. 2001; 71: 123–137.

[23] Li L, Yang SC, Li XL, Zhang FS, Christie P. Interspecific complementary and competitive interactions between intercropped maize and faba bean. Plant Soil 1999; 212: 105–114.

[24] Mei PP, Gui LG, Wang P, Huang JC, Long HY, Christie P, et al Maize/faba bean intercropping with rhizobia inoculation enhances productivity and recovery of fertilizer P in a reclaimed desert soil. Field Crops Res. 2012; 130: 19–27.

[25] Chen Y, Zhou T, Zhang C, Wang K, Liu J, Lu J, et al Rational phosphorus application facilitates the sustainability of the wheat/maize/soybean relays strip intercropping system. PLOS One. 2015 11 5;10(11):e0141725 10.1371/journal.pone.0141725 [PMC free article][PubMed]. 26540207 PMC4634977

[26] EI Dessougi H, Dreele AZ, Claassen N. Growth and phosphorus uptake of maize cultivated alone, in mixed culture with other crops or after incorporation of their residues. J. Plant Nutr. Soil Sci. 2003;166: 254–261.

[27] Yao HY, Bowman D, Wei S. Soil microbial community structure and diversity in a turfgrass chronosequence: land-use change versus turfgrass management. Appl. Soil Ecol. 2006; 34: 209–218.

[28] Carney KM, Matson PA, Bohannan BJM. Diversity and composition of tropical soil nitrifiers across a plant diversity gradient and among land-use types. Ecol. Lett. 2004; 7: 684–694.

[29] Zhang Y, Liu J, Zhang J, Liu H, Liu S, Zhai L, et al Row ratios of intercropping maize and soybean can affect agronomic efficiency of the system and subsequent wheat. PLOS One. 2015 6 10;10(6):e0129245 10.1371/journal.pone.0129245 [PMC free article][PubMed]. 26061566 PMC4463860

[30] Wardle DA, Bardgett RD, Klironomos JN, Setälä H, van der Putten WH, Wall DH. Ecological linkages between aboveground and belowground biota. Science. 2004 6 11; 304(5677):1629–1633. 15192218 10.1126/science.1094875

[31] Song YN, Marschner P, Zhang FS, Bao XG, Li L. Effect of intercropping on bacterial community composition in rhizoshpere of wheat(*Triticum aestivum* L.), maize (*Zea mays* L.) and faba bean (*Vicia faba* L.).Acta Ecol. Sin. 2006; 6: 2268–2274.

[32] Williamsa A, Birkhoferb K, Hedlundb K. Above- and below-ground interactions with agricultural management: Effects of soil microbial communities on barley and aphids. Pedobiologia. 2014; 57: 67–74.

[33] Zhou XG, Yu GB, Wu FZ. Effects of intercropping cucumber with onion or garlic on soil enzyme activities, microbial communities and cucumber yield. Eur. J. Soil Biol. 2011; 47: 279–287.

[34] Du YX, Xie BM, Cai HS, Tang Lu, Guo CH. Structural and functional diversity of rhizosphere microbial community of nine plant species in the Daqing Saline-alkali soil region. Acta Ecologica Sinica,2015-06-12,http://www.cnki.net/kcms/detail/11.2031.Q.20150612.1015.005.html, 10.5846/stxb201404020621

[35] Teaumroong N, Wanapu C, Chankum Y, Arjharn W, Sang-Arthit S, Teaimthaisong K, et al Production and application of bioorganic fertilizers for organic farming systems in Thailand: a case study In: Insam H, Franke-Whittle I, Goberna M.(Eds.), Microbes at Work. Springer, Berlin Heidelberg; 2010 pp. 293–312.

[36] Buyer JS, Teasdale JR, Roberts DP, Zasada IA, Maul JE. Factors affecting soil microbial community structure in tomato cropping systems. Soil Biol. Biochem. 2010; 42: 831–841.

[37] Mar Vazquez M, Cesar S, Azcón R, Barea JM. Interactions between arbuscular mycorrhizal fungi and other microbial inoculants (*Azospirillum*, *Pseudomonas*, *Trichoderma*) and their effects on microbial population and enzyme activities in the rhizosphere of maize plants. Appl. Soil Ecol.2000; 15: 261–272.

[38] Xu Y, Wang G, Jin J, Liu J, Zhang Q, Liu X. Bacterial communities in soybean rhizosphere in response to soil type, soybean genotype, and their growth stage. Soil Biol. Biochem. 2009; 41: 919–925.

[39] Piromyou P, Buranabanyat B, Tantasawat P, Tittabutr P, Boonkerd N, Teaumroong N. Effect of plant growth promoting rhizobacteria (PGPR) inoculation on microbial community structure in rhizosphere of forage corn cultivated in Thailand. Eur. J. Soil Biol. 2011; 47: 44–54.

[40] Marschner P, Crowley D, Yang CH. Development of specific rhizosphere bacterial communities in relation to plant species, nutrition and soil type. Plant Soil. 2004; 261:199–208.

[41] Girvan MS, Bullimore J, Pretty JN, Osborn AM, Ball AS. Soil type is the primary determinant of the composition of the total and active bacterial communities in arable soils. Appl. Environ. Microbiol. 2003; 69: 1800–1809. 12620873 10.1128/AEM.69.3.1800-1809.2003 PMC150080

[42] Li C, Zhang C, Tang L, Xiong Z, Wang B, Jia Z, et al Effect of long-term fertilizing regime on soil microbial diversity and soil property (in Chinese). Acta Microbiologica Sinica. 2014, 54(3):319–329. 24984524

[43] Chu HY, Lin XG, Fujii T, Morimoto S, Yagi K, Hu JL, et al Soil microbial biomass, dehydrogenize activity, bacterial community structure in response to long-term fertilizer management. Soil Biol. Biochem. 2007; 39: 2971–2976.

[44] Nanjareddy K, Blanco L, Arthikala MK, Affantrange XA, Sánchez F, Lara M. Nitrate regulates rhizobial and mycorrhizal symbiosis in common bean (*Phaseolus vulgaris* L.). Journal of integrative plant biology. 2014; 56(3): 281–298. 10.1111/jipb.12156 24387000

[45] Kim YM, Park H, Cho KH, Park JM. Long term assessment of factors affecting nitrifying bacteria communities and N-removal in a full-scale biological process treating high strength hazardous wastewater. Bioresiurce Technology. 2013; 134: 180–189. 10.1016/j.biortech.2013.02.036 23500576

[46] Larkin RP, Honeycutt CW. Effects of different 3-year cropping systems on soil microbial communities and Rhizoctonia diseases of potato. Phytopathology. 2006; 96: 68–79. 10.1094/PHYTO-96-0068 18944206

[47] Zhao J, Ni T, Li Y, Xiong W, Ran W, Shen B, et al Responses of bacterial communities in arable soils in a rice-wheat cropping system to different fertilizer regimes and sampling times. PLOS One. 2014 1 20; 9(1):e85301 10.1371/journal.pone.0085301 [PMC free article][PubMed]. 24465530 PMC3896389

[48] Yang ZP, Hao JM, Miao GY. Utilization of mixture cropping to improve immature loess soil(in chinese). Chin J Appl Environ Biol. 2011; 17 (3): 388–392.

[49] Jia ZH, Yang ZY, Zhang YQ, Miao GY. Study on the quantity of three main colony of soil microbe in wheat farmland(in chinese). Journal of Triticeae Crops. 2004; 24(3): 53–56.

[50] Miao GY, Jia ZH, Yang ZP, Zhang YQ. Quantity difference of rhizosphere microbe of different crops(in chinese). Journal of Shanxi Agricultural University. 2004; 24(2): 93–96.

[51] Xie H, Wang XX, Dai CC, Chen JX, Zhang T. Effects of intercropping peanut with medicinal plants on soil microbial community. Chin. J. Appl. Ecol. 2007; 18: 693–696. 17552215

[52] Marschner P, Yang CH, Lieberei R. Crowley DE. Soil and plant specific effects on bacterial community composition in the rhizosphere. Soil Biol. Biochem. 2001; 33: 1437–1445.

[53] Söderberg KH, Olsson PA, Bååth E. Structure and activity of the bacterial community in the rhizosphere of different plant species and the effect of arbuscular mycorrhizal colonisation. FEMS Microbiol. Ecol. 2002; 40: 223–231. 10.1111/j.1574-6941.2002.tb00955.x 19709230

[54] Lynch JM, Whipps JM. Substrate flow in the rhizosphere. Plant Soil. 1990;129: 1–10.

[55] Barea JM, Werner D, Azco´n-Aguilar C, Azco´ n R. Interactions of arbuscular mycorrhiza and nitrogen fixing symbioses in sustainable agriculture In: Werner D, Newton WE, editors. Agriculture, Forestry, Ecology and the Environment. Kluwer Academic Publishers, The Netherlands; 2005 pp. 199–222.

[56] Badri DV, Vivanco JM. Regulation and function of root exudates. Plant Cell Environ. 2009; 32(6): 666–681. 10.1111/j.1365-3040.2008.01926.x 19143988

[57] Glick BR. The enhancement of plant growth by free-living bacteria. Can. J. Microbiol. 1995; 41: 109–117.

[58] Andersen KS, Winding A. Non-target effects of bacterial biological control agents on soil Protozoa. Biol. Fertil. Soils. 2004; 40: 230–236.

[59] Xiong MB, He JP, Song GY. Effect of root exudations on ecological distribution of rhizospheric microorganisms(in chinese). Chinese Journal of Soil Science, 2002; 33(2): 145–148.

[60] Welbaum GE, Sturz AV, Dong Z, Nowak J. Managing soil microorganisms to improve productivity of agro-ecosystems. Crit. Rev. Plant Sci. 2004; 23: 175–193.

[61] Singh BK, Munro S, Potts JM, Millard P. Influence of grass species and soil type on rhizosphere microbial community structure in grassland soils. Appl. Soil Ecol. 2007; 36: 147–155.

[62] Zhu JP, Cheng K. Effect of organic acids exuded from hydroponic plants roots on nitrogen cycling bacteria(in chinese). Chinese journal of environmental engineering, 2011; 5(9): 2139–2143.

[63] Gao Y, Zhou P, Liang MYZ, Zhang Ch, Shi WJ. Effects of plant species coexistence on soil enzyme activities and soil microbial community structure under Cd and Pb combined pollution. J. Environ. Sci. 2010; 7: 1040–1048. 10.1016/s1001-0742(09)60215-1 21174994

[64] Phillips DA, Streit WR. Modifying rhizosphere microbial communities to enhance nutrient availability in cropping systems. Field Crops Res. 1998; 56: 217–221.

[65] Wang ZG, Jin X, Bao XG, Li XF, Zhao JH, Sun JH, et al Intercropping enhances productivity and maintains the most soil fertility properties relative to sole cropping. PLOS One. 2014 12 8 10.1371/journal.pone.0113984 PMC4259307 25486249

[66] Sun YM, Zhang NN, Wang ET, Yuan HL, Yang JS, Chen WX. Influence of intercropping and intercropping plus rhizobial inoculation on microbial activity and community composition in rhizosphere of alfalfa (*Medicago sativa* L.) and Siberian wild rye (*Elymus sibiricus* L.). FEMS Microbiology ecology. 2009; 70(2): 62–70. 10.1111/j.1574-6941.2009.00752.x 19702874

